# Author Correction: Positive regulation of oxidative phosphorylation by nuclear myosin 1 protects cells from metabolic reprogramming and tumorigenesis in mice

**DOI:** 10.1038/s41467-023-43936-2

**Published:** 2023-11-30

**Authors:** Tomas Venit, Oscar Sapkota, Wael Said Abdrabou, Palanikumar Loganathan, Renu Pasricha, Syed Raza Mahmood, Nadine Hosny El Said, Shimaa Sherif, Sneha Thomas, Salah Abdelrazig, Shady Amin, Davide Bedognetti, Youssef Idaghdour, Mazin Magzoub, Piergiorgio Percipalle

**Affiliations:** 1 Program in Biology, Division of Science and Mathematics, New York University Abu Dhabi (NYUAD), P.O. Box 129188, Abu Dhabi, United Arab Emirates; 2 Center for Genomics and Systems Biology, New York University Abu Dhabi (NYUAD), P.O. Box 129188, Abu Dhabi, United Arab Emirates; 3 Core Technology Platforms, New York University Abu Dhabi (NYUAD), P.O. Box 129188, Abu Dhabi, United Arab Emirates; 4 Translational Medicine Department, Research Branch, Sidra Medicine, Doha, Qatar; 5 Department of Internal Medicine and Medical Specialties (DiMI), University of Genoa, Genoa, Italy; 6 College of Health and Life Sciences, Hamad Bin Khalifa University, Qatar Foundation, Doha, Qatar; 7 Department of Molecular Biosciences, The Wenner-Gren Institute, Stockholm University, SE-106 91 Stockholm, Sweden

**Keywords:** Transcriptomics, Mitochondria, Cancer metabolism

Correction to: *Nature Communications* 10.1038/s41467-023-42093-w, published online 10 October 2023

In the original version of this Article, there were errors in Fig. 2g and h. In both cases, the asterisks denoting statistical significance were inadvertently misaligned with the brackets indicating the categories being compared. These errors have now been corrected in both the PDF and HTML versions of the Article.

