# Supplementary material for: Positive regulation of oxidative phosphorylation by nuclear myosin 1 protects cells from metabolic reprogramming and tumorigenesis in mice
Source: Nat Commun. 2023 Oct 10;14:6328. doi: 10.1038/s41467-023-42093-w (PMC10564744; doi:10.1038/s41467-023-42093-w)
Supplement: Supplementary file 1 — Supplementary Information [file 41467_2023_42093_MOESM1_ESM.pdf]

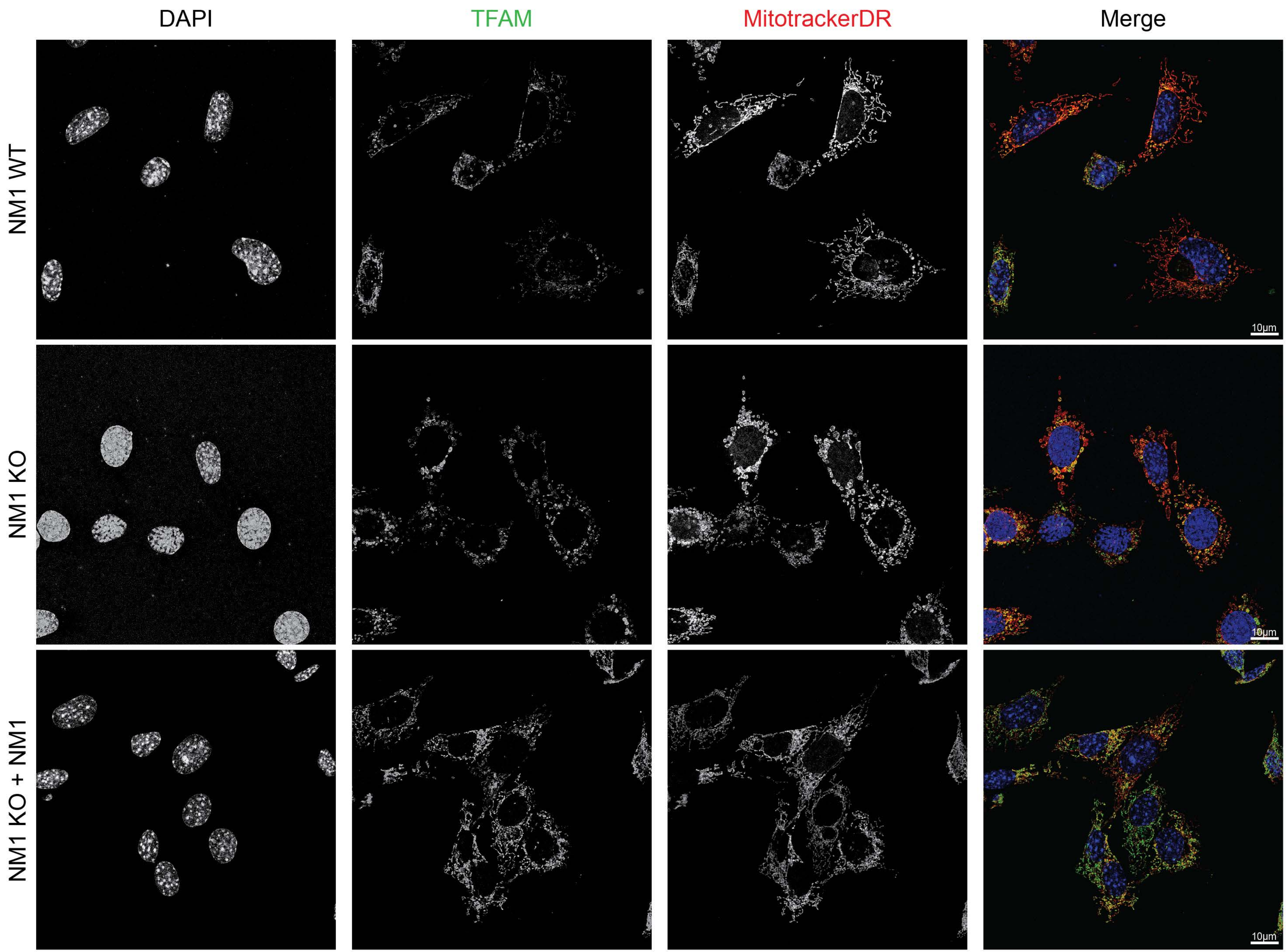

Supplementary figure 1. **Confocal microscopy of IF stained WT, KO and KO+NM1 cells.** Representative confocal microscopy images of MitoTracker DR and TFAM stained mitochondria in WT, KO and KO+NM1 MEFs. Scale bar is 10µm.

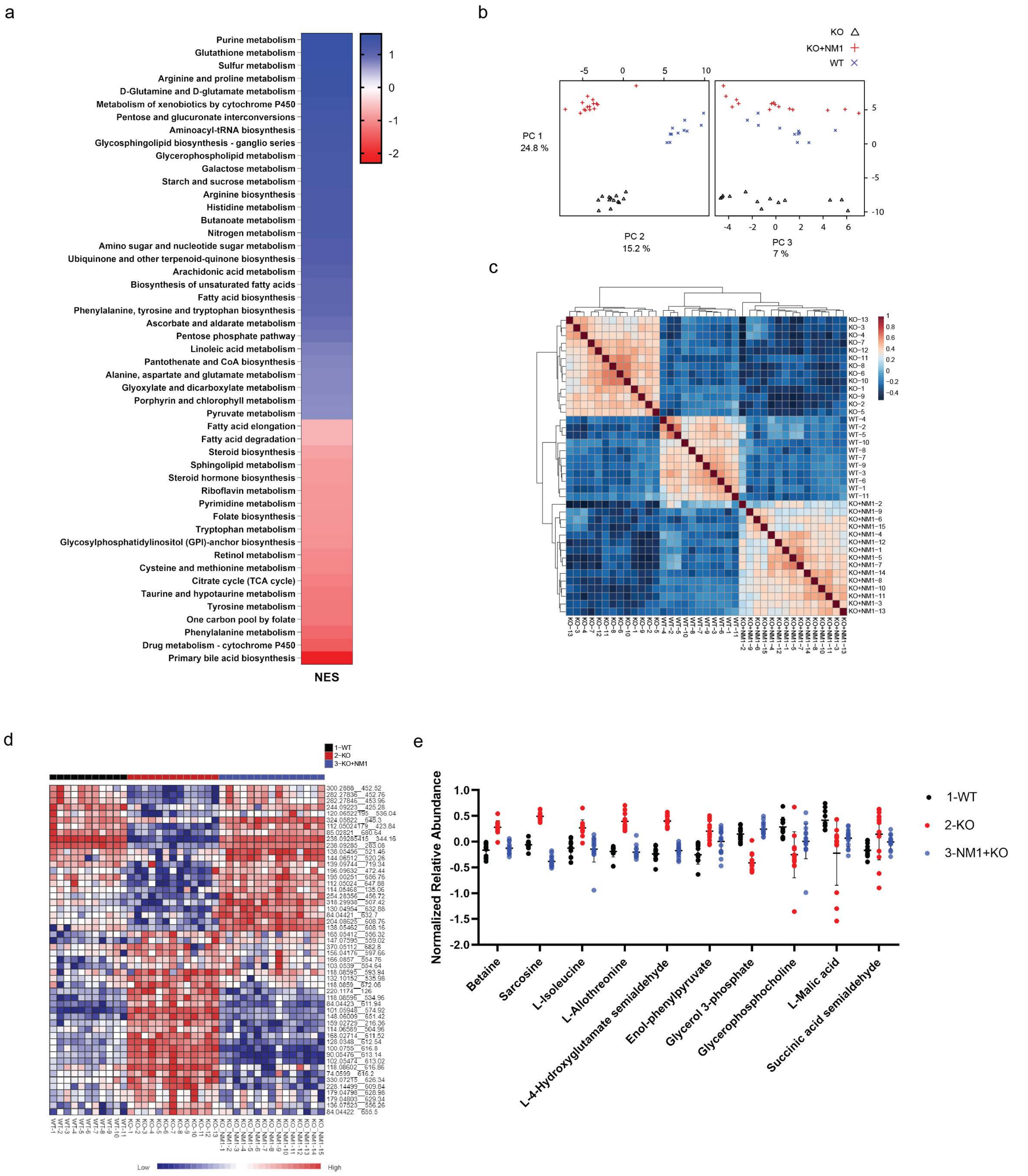

Supplementary figure 2. **Metabolomic profiling of NM1 WT, KO and KO+NM1 cells. a** GSEA analysis of metabolites identified using negative ionization. Affected metabolic pathways are organized descending based on their normalized enrichment scores (NES) where blue represents pathways that are overrepresented and red pathways that are underrepresented in NM1 KO Cells. **b** PCA analysis of metabolomic data was performed on the normalized peak dataset from WT, KO and KO+NM1 cells. **c** Global metabolomic correlation matrix clustering based on similarity from WT, KO and KO+NM1 cells. **d** Heatmap of 52 metabolic features identified by a functional mummichog pathway enrichment analysis. **e** Graph represents the normalized relative abundance of randomly selected identified compounds in metabolomic screen. center line represents mean value and error bars represents standard deviation. n=11 for WT, n=13 for KO, n=15 for KO+NM1. Source data are provided as a Source Data file.

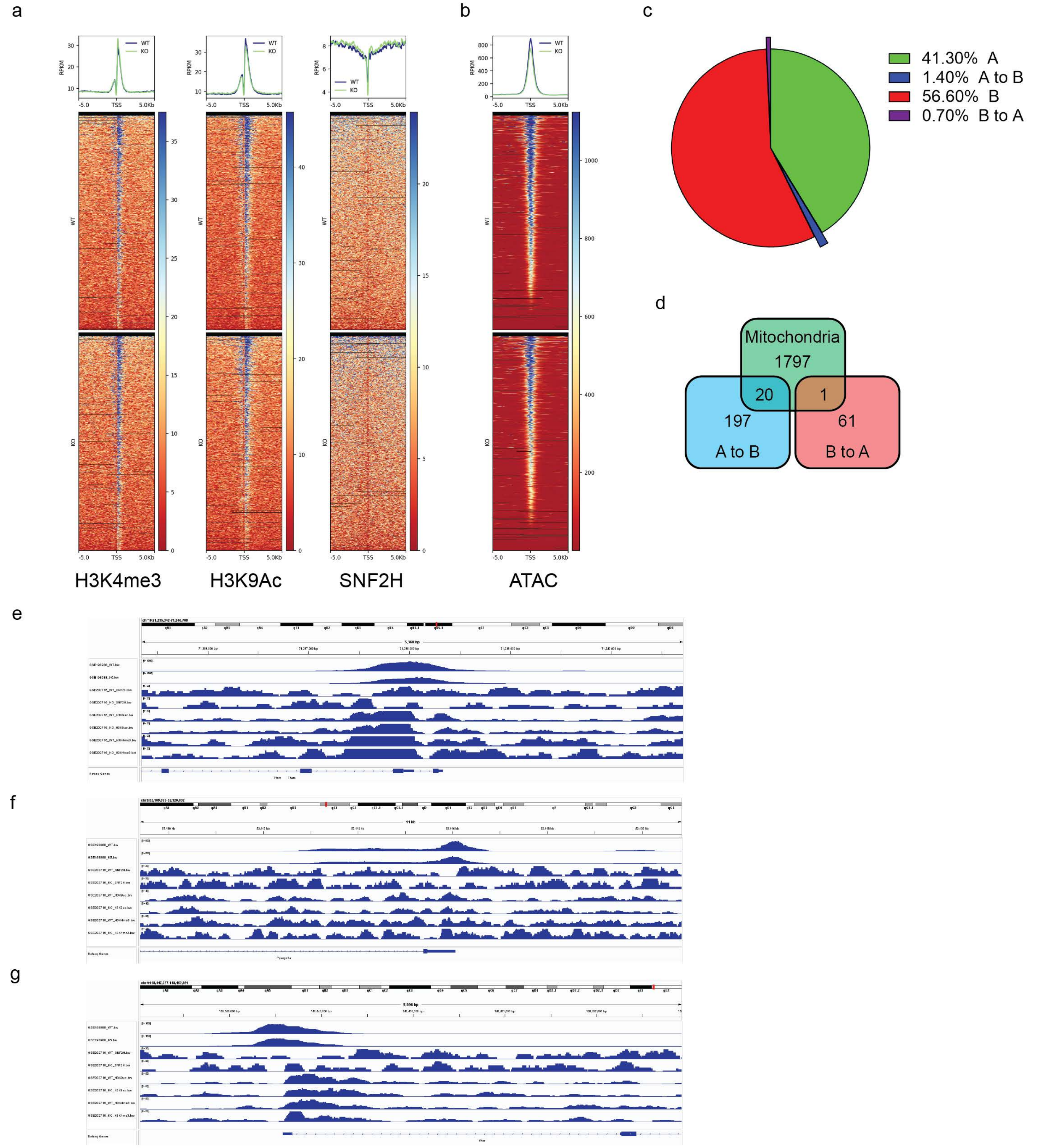

Supplementary figure 3. **ATAC-seq, ChIP-seq, and HiC-Seq analysis of NM1 WT and KO cells. a** Profile plots showing average ChIP signal intensity (top) and heatmaps showing scaled ChIP read density (bottom) of H3K4me3, H3K9Ac, and SNF2H around mitochondrial gene transcription start sites in WT and KO cells. The scale bar shows normalized RPKM. **b** Profile plot showing average ATAC signal intensity (top) and heatmaps showing scaled ATAC read density (bottom) around mitochondrial gene transcription start sites in WT and KO cells. The scale bar shows normalized RPKM. **c** Pie chart showing the distribution of switching and stable compartments at 500kb resolution in NM1 KO vs WT cells. **d** Venn diagram showing the distribution of mitochondrial genes in switching compartments. **e** ATAC-seq and ChIP-seq profiles for SNF2H, H3K9ac and H3K4me3 marks along TSS of TFAM gene in WT and KO cells. **f** ATAC-seq and ChIP-seq profiles for SNF2H, H3K9ac and H3K4me3 marks along TSS of PGC1α gene in WT and KO cells. **g** ATAC-seq and ChIP-seq profiles for SNF2H, H3K9ac and H3K4me3 marks along TSS of mTOR gene in WT and KO cells. Source data are provided as a Source Data file.
