# Supplementary material for: Positive regulation of oxidative phosphorylation by nuclear myosin 1 protects cells from metabolic reprogramming and tumorigenesis in mice
Source: Nat Commun. 2023 Oct 10;14:6328. doi: 10.1038/s41467-023-42093-w (PMC10564744; doi:10.1038/s41467-023-42093-w)
Supplement: Supplementary file 3 — Description of Additional Supplementary Files [file 41467_2023_42093_MOESM3_ESM.pdf]

Description of Additional Supplementary Files

File name: Supplementary Data 1

Description: Predicted pathways (human)

File name: Supplementary Data 2

Description: Primers used in the study

File name: Supplementary Data 3

Description: TCGA Regression analyses

File name: Supplementary Data 4

Description: TCGA Regression analyses

File name: Supplementary Data 5

Description: TCGA Regression analyses

File name: Supplementary Data 6

Description: TCGA Regression analyses

File name: Supplementary Data 7

Description: TCGA Regression analyses
