# Supplementary material for: Positive regulation of oxidative phosphorylation by nuclear myosin 1 protects cells from metabolic reprogramming and tumorigenesis in mice
Source: Nat Commun. 2023 Oct 10;14:6328. doi: 10.1038/s41467-023-42093-w (PMC10564744; doi:10.1038/s41467-023-42093-w)
Supplement: Supplementary file 11 — Reporting Summary [file 41467_2023_42093_MOESM11_ESM.pdf]

nature portfolio

Corresponding author(s): *Double-anonymous peer review submissions: write DAPR and your manuscript number here instead of author names.*

Last updated by author(s): *YYYY-MM-DD*

Reporting Summary

Nature Portfolio wishes to improve the reproducibility of the work that we publish. This form provides structure for consistency and transparency in reporting. For further information on Nature Portfolio policies, see our Editorial Policies and the Editorial Policy Checklist.

Statistics

For all statistical analyses, confirm that the following items are present in the figure legend, table legend, main text, or Methods section.

n/a	Confirmed	
☐	☒	The exact sample size (*n*) for each experimental group/condition, given as a discrete number and unit of measurement
☐	☒	A statement on whether measurements were taken from distinct samples or whether the same sample was measured repeatedly
☐	☒	The statistical test(s) used AND whether they are one- or two-sided<br>*Only common tests should be described solely by name; describe more complex techniques in the Methods section.*
☒	☐	A description of all covariates tested
☒	☐	A description of any assumptions or corrections, such as tests of normality and adjustment for multiple comparisons
☐	☒	A full description of the statistical parameters including central tendency (e.g. means) or other basic estimates (e.g. regression coefficient) AND variation (e.g. standard deviation) or associated estimates of uncertainty (e.g. confidence intervals)
☐	☒	For null hypothesis testing, the test statistic (e.g. *F*, *t*, *r*) with confidence intervals, effect sizes, degrees of freedom and *P* value noted<br>*Give P values as exact values whenever suitable.*
☒	☐	For Bayesian analysis, information on the choice of priors and Markov chain Monte Carlo settings
☒	☐	For hierarchical and complex designs, identification of the appropriate level for tests and full reporting of outcomes
☒	☐	Estimates of effect sizes (e.g. Cohen's *d*, Pearson's *r*), indicating how they were calculated

Our web collection on statistics for biologists contains articles on many of the points above.

Software and code

Policy information about availability of computer code

Data collection	Data collection is described in the subsequent part separately for each method.
Data analysis	Large scale Ultra-Performance Liquid Chromatography High-Resolution Mass Spectrometry<br>5 replicates of NM1 WT and KO cells were used for metabolomic profiling. Overnight grown cells (approximately 3x105 per sample) were washed twice with ice-cold 0.9% NaCl solution and 300µl of 100% Methanol was added to each well. After 3 minutes of incubation on ice, cells were scraped using a precooled cell scraper and moved to a cold Eppendorf tube. Cell extracts were spun down at 15000rpm for 15 min at 4°C and 200µl of each supernatant was moved to a new tube for further processing. Next, samples were vacuum dried and reconstituted in 200µl of cyclohexane/water (1:1) solution for subsequent analysis by Ultra Performance Liquid Chromatography High-Resolution Mass Spectrometry (UPLC-HRMS) performed at the VIB Metabolomics core facility (Belgium). 10 ul of each sample were injected on a Waters Acquity UHPLC device connected to a Vion HDMS Q-TOF mass spectrometer. Chromatographic separation was carried out on an ACQUITY UPLC BEH C18 (50 × 2.1 mm, 1.7 µm) column from Watersunder under the constant temperature of 40°C. A gradient of two buffers was used for separation: buffer A (99:1:0.1 water:acetonitrile:formic acid, pH 3) and buffer B (99:1:0.1 acetonitrile:water: formic acid, pH 3), as follows: 99% A for 0.1 min decreased to 50% A in 5 min, decreased to 30% from 5 to 7 minutes, and decreased to 0% from 7 to 10 minutes. The flow rate was set to 0. 5 mL min−1. Both positive and negative Electrospray Ionization (ESI) was applied to screen for a broad array of chemical classes of metabolites present in the samples. The LockSpray ion source was operated in positive/negative electrospray ionization mode under the following specific conditions: capillary voltage, 2.5 kV; reference capillary voltage, 2.5 kV; source temperature, 120°C; desolvation gas temperature, 600°C; desolvation gas flow, 1000 L h−1; and cone gas flow, 50 L h−1. The collision energy for the full MS scan was set at 6 eV for low energy settings, for high energy settings (HDMSe) it was ramped from 28 to 70 eV. Mass range was set from 50 to 1000Da, scan time was set at 0.1s. Nitrogen (greater than 99.5%) was employed as desolvation and cone gas. Leucine-enkephalin (250 pg/µL solubilized in water:acetonitrile 1:1 [v/v], with 0.1% formic acid) was used for the lock mass calibration, with scanning every 1 min at a scan time of 0.1 s. Profile data was recorded through Unifi Workstation v2.0 (Waters).

Data normalization was performed to remove potential variation resulting from instrument inter-run tuning differences. Raw MS peak data representing the abundance of each detected compound were subject to median standardization and missing values were imputed by the minimum value. Compounds with missing values in more than 50% of samples were considered missing. Standardized data that passed the quality control step were then log2 transformed and IQR normalized using JMP Genomics v8 (SAS Institute, Cary, NC) to remove potential technical artifacts and outliers. PCA and hierarchical clustering were done to explore the correlation structure in the data across the two conditions (WT and KO).

Functional and metabolic pathway enrichment analysis of large-scale Ultra-Performance High-Resolution LC-MS

Functional analysis of curated normalized peak data was performed using MetaboAnalyst v5.0 using an existing protocol 97. Implemented Gene Set Enrichment Analysis (GSEA) method in the Functional Analysis module of MetaboAnalyst v5.0 (Accessed in 2022 from http://www.metaboanalyst.ca/) was used to identify sets of functionally related compounds and evaluate their enrichment of potential functions defined by metabolic pathways. GSEA analysis of compounds identified using positive and negative ionization was performed separately. Putative annotation of MS peaks data considering different adducts and ion modes was performed. m/z values and retention time dimensions both were used to increase confidence in identifying compounds and improve the accuracy of functional interpretations. Annotated compounds were then mapped onto Mus musculus (mouse) [KEGG] 98 and a curated 912 metabolic data sets predicted to change due to dysfunctional enzymes based on human metabolism, separately 97, for pathway activity prediction (Supplementary table 1). GSEA calculates the Enrichment score (ES) by walking down a ranked list of metabolites, increasing a running-sum statistic when a metabolite is in the metabolite set and decreasing it when it is not. A metabolite set is defined in this context as a group of metabolites with collective biological functions or common behaviors, regulations, or structures. In this method, the ES of each enriched pathway is calculated to reflect the degree to which a metabolite set is overrepresented at the top or bottom of a ranked list of metabolites. Each ES is then normalized by the average of all ES scores against all permutations of the expression dataset to generate normalized enrichment scores (NES) that are used to compare analysis results across metabolite sets. By normalizing the enrichment score, GSEA accounts for differences in metabolite set size and correlations between metabolite sets and the expression dataset. A positive NES indicates metabolite set enrichment at the top of the ranked list; a negative NES indicates metabolite set enrichment at the bottom of the ranked list 99. Finally, compound hits were identified for each enriched pathway. Raw metabolomic data are publicly available in the Mendeley database (http://dx.doi.org/10.17632/nxzs4dtztg.1; https://data.mendeley.com/datasets/nxzs4dtztg/2).

Small scale Liquid Chromatography Mass Spectrometry

A Blank (n=3) and the WT, KO and KO+NM1 extracted samples (randomised) were analysed in a single analytical run using simultaneous ESI positive and negative ion modes with LC-MS. Quality control (QC) sample was prepared by pooling equal volumes of 20 µL from each sample in the study, vortexed for 30 s and centrifuged at 13,000 g, at 4 ºC for 5 min. Six injections of the pooled QC sample were analysed at the beginning of the sample run to equilibrate the column prior to the analysis of the samples. Pooled QC injections were interspaced throughout the run to check the stability, robustness, repeatability and performance of the analytical system. QC sample were also used for the identification of metabolites in the study. Chromatography was performed using Vanquish UHPLC system (Thermo Fisher Scientific, Waltham, MA, USA) on a Kinetex HILIC column (2.1 × 100 mm, 2.6 µm particle size, 100 Å, Phenomenex, Torrance, CA, USA). The column was maintained at 30 °C and a flow rate of 200 µL/min. Mobile phases used were: (A) 0.1% formic acid, 10 mM ammonium formate in water and (B) 0.1% formic acid in acetonitrile. The gradient started with 5% (A) and increased to 60% (A) over 10.5 min and held at 60% (A) for another 4.5 min. The composition was returned to its initial conditions of 5% (A) in 2 min. The flow rate was then increased to 300 µL/min in 1 min and the column was left to re-equilibrate for 12.5 min before return to its initial flow rate of 200 L/min in 2 min (32 min total time). The injection volume was 5 µL and samples were maintained at 4 °C during the analysis. Orbitrap Fusion Lumos Tribrid Mass Spectrometer (Thermo Fisher Scientific, Waltham, MA, USA) was used in simultaneous ESI+ and ESI- modes for full LC-MS profiling and to generate data dependent MS/MS (ddMS/MS) accurate mass spectra for identification (most intense peaks, TopN). The operational parameters were: spray voltage 3.5 kV (ESI+), 3.5 kV (ESI-), sheath, auxiliary and sweep gas flow rate were: 45, 8 and 1 (arbitrary units), respectively, for both modes. Capillary and heater temperature were maintained at 350 ºC and 300 ºC, respectively. Data were acquired in full scan mode with resolution 60,000 from m/z 50-1000. TopN ddMS/MS scans were performed on the QC sample at a resolution of 17,500 and stepped collision energies (CE) of 20, 35 and 50.

The raw LC–MS dataset of the samples in the study were initially processed with Compound Discoverer 3.3 (Thermo Fisher Scientific, Waltham, MA, USA) for untargeted peak-picking, peak alignment, peak deconvolution and annotation of related peaks using a tailored untargeted metabolomics workflow. The generated raw LC-MS peaks were subjected to a QC correction approach in which the detected peaks must be present in ≥ 30% of the QC sample to be considered sample related peaks for further analysis. The dataset was then normalised to constant median and Log transformed to restore normality with 891 metabolic features (mz/rt) detected in all samples.

Functional analysis of small-scale LC-MS

Data consisted of 891 metabolic features (mz/rt) were detected in 39 samples (11 WT, 13 KO and 15 KO+WT). The peak data for the metabolic features were normalized by the sample median, log transformed (base 10) and scaled using pareto scaling (mean-centered and divided by the square root of standard deviation of each variable) using MetaboAnalyst v5.0 using an existing protocol97. Implemented mummichog pathway Enrichment Analysis method in the Functional Analysis module of MetaboAnalyst v5.0 (Accessed in 2023 from http://www.metaboanalyst.ca/) was used to identify sets of functionally related compounds and evaluate their enrichment of potential functions that are potentially rescued in the experiment. The analysis leverages the power of known metabolic pathways to gain functional insight directly from m/z features and retention times. The mummichog algorithm includes: 1) Permutations: A list of metabolites (the same length as the number of significant m/z features) are inferred from the set of m/z features, considering all potential matches (isotopes/adducts). Raw metabolomic data are publicly available in the Mendeley database (http://dx.doi.org/10.17632/nxzs4dtztg.1; https://data.mendeley.com/datasets/nxzs4dtztg/2).

HiC-Seq sample preparation and analysis

NM1 WT and KO cells were grown under standard conditions to 80% confluency with two biological replicates used for each condition. After trypsinization, cells were washed twice in 1x PBS buffer and 1 million cells per condition were fixed with 2% formaldehyde for 10 mins. The cell pellets were washed twice by 1× PBS, fast-frozen on dry ice, and stored at −80 °C. All subsequent processing and Hi-C were performed by Genome Technology Center at NYU Langone Health, NY, using standard DNA extraction and library preparation protocols and the Arima Hi-C kit (Arima Genomics, San Diego, CA), respectively. Subsequently, sequencing libraries were prepared by using a modified version of the KAPA HyperPrep library kit (KAPA Biosystems, Willmington, MA) and sequenced by a NovaSeq instrument. Raw sequencing data were processed using the HiCUP 100 pipeline and analyzed using HOMER 101 (http://homer.ucsd.edu/homer/). For preprocessing with HiCUP, a digest file compatible with the Arima protocol was produced with HiCUP digester using the option –arima. Processed bam files produced by HiCUP were converted to HOMER format using the script hicup2homer followed by conversion to homer tag directories using the command makeTagDirectory -format HiCsummary. PCA analysis was performed using HOMER with the command runHiCpca.pl -genome mm10 -res 500000 -window 500000 followed by annotation and differential analysis with the scripts annotatePeaks.pl and getDiffExpression.pl. Bins changing PC1 values from positive to negative or vice versa with an FDR of less than 0.05 between WT and KO cells were classified as switching. Protein-coding genes with TSS overlapping each compartment were identified using the valr package 102. TADs were called on replicate-merged tag directories of WT-MEFs using the HOMER function findTADsAndLoops.pl with -window and -res set to 50000. The insulation score of the identified domains in all replicates of each condition was extracted using the command findTADsAndLoops.pl with the

“score” option followed by differential analysis using getDiffExpression.pl. Domains showing the change in insulation score with an adjusted p-value less than 0.05 were classified as a differential. Data is available in the Gene Expression Omnibus (GEO) database under accession number GSE198989 (https://www.ncbi.nlm.nih.gov/geo/query/acc.cgi?acc=GSE198989).

ATAC-Seq sample preparation and analysis

NM1 WT and KO cells were used for the analysis performed commercially by Novogene (Beijing, China) with two biological replicates used for each condition. Isolated cell nuclei were mixed with Tn5 Transposase with two adapters for 30 min at 37 °C for DNA fragmentation, followed by DNA purification and amplification with a limited PCR cycle using index primers. Libraries were prepared according to recommended Illumina protocols and sequenced by NovaSeq 6000 instrument. ATAC-Seq processing and quality control were performed by Novogene (Beijing, China). Adapter trimming on raw fastq files was performed using trim-galore with default settings. Surviving paired reads were aligned against the relevant reference genome (GRCm38) using Burrows-Wheeler Aligner BWA-MEM. The Resulting BAM alignments were cleaned, sorted, and deduplicated (PCR and Optical duplicates) with PICARD tools (http://broadinstitute.github.io/picard). Processed bam files were converted to HOMER tag directories followed by annotation and differential analysis with the scripts annotatePeaks.pl and getDiffExpression.pl. ATAC-Seq peaks were called on cleaned, deduplicated bam files of both replicates of each condition together using macs2 with the parameters -q 0.05 -g mm/hg --keep-dup all --nomodel --shift −100 --extsize 200 -B --broad -f BAMPE. Peaks of the two conditions being compared were merged using homer command mergePeaks and annotated with annotatePeaks.pl. Differential peaks were identified using the standard DESeq2 pipeline with lfcThreshold =1.5 and alpha=0.05. Data is available in the Gene Expression Omnibus (GEO) database under accession number GSE198988 (https://www.ncbi.nlm.nih.gov/geo/query/acc.cgi?acc=GSE198988).

ChIP-Seq sample preparation and analysis

Cells were crosslinked (two biological replicates per condition and input controls) using 1% formaldehyde for 10 min followed by quenching with 0.125 M Glycine for 5 min and lysis with lysis buffer 1 (50 mM Hepes KOH pH 7.5, 10 mM NaCl, 1 mM EDTA, 10% glycerol, 0.5% NP-40, 0.25% Triton X-100). Nuclei were pelleted, collected, and washed using lysis buffer 2 (10 mM Tris-HCl pH 8, 200 mM NaCl, 1 mM EDTA, 0.5 mM EGTA). This was followed by lysis using lysis buffer 3 (10 mM Tris-HCl pH 8; 100 mM NaCl, 1 mM EDTA; 0.5 mM EGTA; 0.1% Na-Deoxycholate, 0.5% N-laurylsarcosine). Chromatin was sheared using Qsonica Sonicator (4 cycles of 3 min at 70 % Amplitude), and then checked on 0.8% agarose gel. 100 μg of fragmented chromatin was mixed with the appropriate antibody. The protein-antibody immunocomplexes were recovered by the Pierce Protein A/G Magnetic Beads. Beads and attached immunocomplexes were washed twice using Low salt wash buffer (0.1% SDS; 2 mM EDTA, 1% Triton X-100, 20 mM Tris-HCl pH 8, 150 mM NaCl), and High Salt wash buffer (0.1% SDS, 2 mM EDTA, 1% Triton X-100, 20 mM Tris-HCl pH 8, 500 mM NaCl), respectively. The beads were resuspended in elution buffer (50 mM Tris-HCl pH 8, 10 mM EDTA, 1% SDS). De-crosslinking was achieved by adding 8 μL of 5 M NaCl and incubating at 65 °C overnight. RNase A (1 μL 10 mg/mL) was added for a 30 min incubation at 37 °C. Then, 4 μL 0.5 M EDTA, 8 μL 1 M Tris-HCl, and 1 μL 20 mg/mL proteinase K (0.2 mg/mL) were added for a 2-hour incubation at 42 °C to digest the chromatin. DNA was purified by a QIAquick PCR purification kit for qPCR analysis and sequencing. Raw reads were quality trimmed using Trimmomatic 103 and analyzed with FastQC (http://www.bioinformatics.babraham.ac.uk/projects/fastqc) to trim low-quality bases, systematic base-calling errors, and sequencing adapter contamination. Specific parameters used were “trimmomatic_adapter.fa:2:30:10 TRAILING:3 LEADING:3 SLIDINGWINDOW:4:15 MINLEN:36”. Surviving paired reads were then aligned against the mouse reference genome (GRCm38) using Burrows-Wheeler Aligner BWA-MEM. The resulting BAM alignments were cleaned, sorted, and deduplicated (PCR and Optical duplicates) with PICARD tools (http://broadinstitute.github.io/picard). Bigwig files were generated using deeptools 104 command bamCoverage -bs 10 -e --ignoreDuplicates –normalizeUsingRPKM after excluding encode blacklisted regions. Bigwig files were analyzed with the computeMatrix function of deeptools to plot the average signal around regions of interest. Peaks were called using macs2 105 on replicate-merged bam files with relevant input control and q=0.05. The –broad flag was used for H3K27me3 while narrow peaks were called for other epigenetic marks. Peaks of the two conditions being compared were merged using homer command mergePeaks followed by annotation and differential analysis with the scripts annotatePeaks.pl and getDiffExpression.pl. Peaks showing differential expression with an adjusted p-value less than 0.05 were classified as differentially expressed. Data is available in the Gene Expression Omnibus (GEO) database under accession number GSE202716 (https://www.ncbi.nlm.nih.gov/geo/query/acc.cgi?acc=GSE202716).

RNA-Seq library preparation, sequencing, and analysis

For transcriptional profiling of tumor tissues, tumor derived from KO cells and tumor derived from KO+NM1 cells were selected for comparison. Each tumor was divided into equal pieces and each piece was separately homogenized in RNAzol reagent by using the Bead Ruptor 96 Homogenizer (Omni International). The RNA-Seq library was prepared by using the NEBNext Ultra II RNA Library Prep Kit for Illumina (NEB) and sequenced with the NextSeq 500/550 sequencing platform (performed at the NYUAD Sequencing Center). All of the subsequent analysis, including quality trimming, was executed using the BioSAILs workflow execution system. Trimmomatic (version 0.36) was used for quality trimming of the raw reads to get rid of low-quality bases, systematic base-calling errors, as well sequencing adapter contamination 103. The quality of the sequenced reads pre/post quality trimming was assessed by FastQC and only the reads that passed quality trimming in pairs were retained for downstream analysis (https://www.bioinformatics.babraham.ac.uk/projects/fastqc/). The quality-trimmed RNA-Seq reads were aligned to the Mus musculus GRCm38 (mm10) genome using HISAT2 (version 2.0.4) 108. The conversion and sorting of SAM alignment files for each sequenced sample to BAM format were done by using SAMtools (version 0.1.19) 109. The BAM alignment files were processed using HTseq-count, using the reference annotation file to produce raw counts for each sample. The raw counts were then analyzed using the online analysis portal NASQAR (http://nasqar.abudhabi.nyu.edu/), to merge, normalize, and identify differentially expressed genes (DEG). DEG by at least twofold (log2(FC) ≥ 1 and adjusted p-value of <0.05 for upregulated genes, and log2(FC) ≤ −1 and adjusted p-value of <0.05 for downregulated genes) between the tumors derived from KO and KO+NM1 were subjected to GO enrichment using DAVID Bioinformatics (https://david.ncifcrf.gov/) 110. RNA-Seq data were deposited in the Gene Expression Omnibus (GEO) database under accession number GSE236679 (https://www.ncbi.nlm.nih.gov/geo/query/acc.cgi?acc=GSE236679).

The RNA-Seq data set of NM1 WT and KO primary mouse embryonic fibroblast used in this study (Figure 1D, 1E, and 1F), was described previously 23 and is available in the Gene Expression Omnibus (GEO) database under accession number GSE133506 (https://www.ncbi.nlm.nih.gov/geo/query/acc.cgi?acc=GSE133506). The parameters and subsequent analysis used to define differentially expressed genes were the same as for RNA-seq analysis of tumor tissues. The list of genes used for comparative analysis was based on GO terms “oxidative phosphorylation” (GO:0006119) and “mitochondrion” (GO:0005739) (http://geneontology.org) 111,112.

COSMIC database

Mutagenesis rate and expression status comparisons of Myo1C, p53 and mTOR in human cancer samples are based on the data downloaded from COSMIC - Catalogue of Somatic Mutations in Cancer (version COSMIC v96) 62.

TCGA RNAseq data

All analyses on the TCGA data were performed using R (v 4.2.1). The RNAseq STAR counts data were downloaded and processed using Biolinks (v 2.24.3) 113. The RNAseq STAR counts data for 32 solid cancer types were downloaded from the GDC portal (https://portal.gdc.cancer.gov/repository) and processed using Biolinks (v 2.24.3) 113. TCGA primary tumor samples were extracted using TCGA-Assembler 2 (v 2.0.3) 114. For skin cutaneous melanoma (SKCM), the primary tumor sample is frequently unavailable for genomic analysis and TCGA mostly includes lymph nodes metastatic samples, which were also included in the analysis. In case of duplicate aliquots/samples, one was removed randomly. Only one sample for patient was included. Gene symbols were converted to official HGNC gene symbols. Gene expression normalization was performed within lanes, to correct for gene-specific effects (including GC-content) and between lanes, to correct for sample-related differences (including sequencing depth) using EDASeq (Exploratory Data Analysis and Normalization for RNASeq) (v 2.12.0) 115. The resulting

expression values were quantile normalized using preprocessCore (v 1.36.0) 116.
TCGA somatic mutation data
Somatic mutation calls from the TCGA MC3 Project were downloaded using TCGAmutations (https://github.com/PoisonAlien/TCGAmutations) 117. The MAF file was filtered to only include non-synonymous mutations (“Frame_Shift_Del”, “Frame_Shift_Ins”, “In_Frame_Del”, “In_Frame_Ins”, “Missense_Mutation”, “Nonsense_Mutation”, “Splice_Site”, “Translation_Start_Site”, “Nonstop_Mutation”). Samples were stratified into two groups: with and without non-synonymous mutations in the Myo1C gene. Tumor samples with both mutations and RNAseq data available were used for the analysis. These include samples from the following tumor types: Uterine Corpus Endometrial Carcinoma (UCEC; n = 528), Colon Adenocarcinoma (COAD; n= 402), Skin Cutaneous Melanoma (SKCM; n= 465), Stomach adenocarcinoma (STAD; n = 372), Head and Neck Squamous Cell Carcinoma (HNSC; n= 495), Lung Adenocarcinoma (LUAD; n= 511), Breast Invasive Carcinoma (BRCA; n= 1017) , Bladder Urothelial Carcinoma (BLCA; n= 405), Kidney Renal Papillary Cell Carcinoma (KIRP; n = 280) , Brain Lower Grade Glioma (LGG; n= 509), Liver Hepatocellular Carcinoma (LIHC; n= 358), Lung Squamous Cell Carcinoma (LUSC; n= 482), Thyroid Carcinoma (THCA; n= 488), Cervical Squamous Cell Carcinoma and Endocervical Adenocarcinoma (CESC; n= 286), Esophageal Carcinoma (ESCA; n= 161) , Ovarian Serous Cystadenocarcinoma (OV; n= 257), Cholangiocarcinoma (CHOL; n= 35), Glioblastoma Multiforme (GBM; n= 150), Kidney Renal Clear Cell Carcinoma (KIRC; n= 366), Pancreatic Adenocarcinoma (PAAD; n= 168), Prostate Adenocarcinoma (PRAD; n= 493), Sarcoma (SARC; n= 234) , Uveal Melanoma (UVM; n= 80), Adrenocortical Carcinoma (ACC; n= 79), Lymphoid Neoplasm Diffuse Large B-cell Lymphoma (DLBC; n = 37), Kidney Chromophobe (KICH; n= 65), Mesothelioma (MESO; n = 82), Pheochromocytoma and Paraganglioma (PCPG; n= 178), Testicular Germ Cell Tumors (TGCT; n= 144), Thymoma (THYM; n= 119), Uterine Carcinosarcoma (UCS; n= 57), Rectum Adenocarcinoma (READ; n= 145). Altogether, there were 9448 samples available for the Pan-cancer analysis.
TCGA Oncogenic pathway enrichment score
Gene sets that reflect cancer-cell intrinsic (oncogenic) pathways were selected from multiple sources as described in detail in Roelands et al 118. After removing redundant pathways, 43 oncogenic pathways were included in the final analysis. Single sample gene set enrichment analysis (ssGSEA) was applied to the log2 transformed, normalized gene expression matrix, using GSVA (v 1.44.5) 119, to generate the pathway enrichment score (ES) for each sample.
TCGA Regression analyses
Linear regression models were fit to define the association between Myo1C non-synonymous mutations and the oncogenic pathways’ ES using the lm function. This analysis was performed in the entire dataset (Pan-cancer, n= 9448) as well as within each cancer type separately (Per-cancer). In the Pan-cancer analysis we used the Benjamini-Hochberg FDR to correct for multiple hypothesis testing. Cancer types with less than three samples harboring Myo1C non-synonymous mutations (ACC, GBM, KIRC, KICH, MESO, SARC, DLBC, READ, PCPG, TGCT, CESC, THYM, ESCA, UCS, PAAD, PRAD, CHOL, UVM) were excluded from the Per-cancer analysis but retained in the Pan-cancer analysis. The results were visualized using ComplexHeatmap (v.2.1.2) 120 and ggplot2 (v.3.4.1) 121. Associations with an FDR < 0.1 in the Pan-cancer analyses were plotted. Data used are extracted from an open-access repository (https://portal.gdc.cancer.gov/repository). All data relevant to the study are included in the article (Supplementary data 3-7)).
Statistics and reproducibility
GraphPad Prism 8.3.0 software was used for statistical analysis. Unpaired t-test was used in all the experiments, unless otherwise stated in the text. Error bars in boxplots represents minimum and maximum values. Error bars in bar charts represents SD. Statistical significance is marked with *p <0.05, **p < 0.01, ***p < 0.001, ****p < 0.0001, ns (not significant). Each experiment was performed at least in triplicates. Exact description of sample sizes and number of replicates is defined for each experimental procedure in Methods.

For manuscripts utilizing custom algorithms or software that are central to the research but not yet described in published literature, software must be made available to editors and reviewers. We strongly encourage code deposition in a community repository (e.g. GitHub). See the Nature Portfolio guidelines for submitting code & software for further information.

Data

Policy information about availability of data

All manuscripts must include a data availability statement. This statement should provide the following information, where applicable:

- Accession codes, unique identifiers, or web links for publicly available datasets
- A description of any restrictions on data availability
- For clinical datasets or third party data, please ensure that the statement adheres to our policy

Data Availability
Source data for the majority of experiments are provided as a Source Data file. Tumor RNA-Seq data were deposited in the Gene Expression Omnibus (GEO) database under accession number GSE236679. The RNA-Seq data set of NM1 WT and KO primary mouse embryonic fibroblast used in this study (Figure 1D, 1E, and 1F), was described previously 23 and is available in the Gene Expression Omnibus (GEO) database under accession number GSE133506. Hi-C data is available in the Gene Expression Omnibus (GEO) database under accession number GSE198989. ATAC-Seq data is available in the Gene Expression Omnibus (GEO) database under accession number GSE198988. ChIP-Seq data is available in the Gene Expression Omnibus (GEO) database under accession number GSE202716. Raw metabolomic data from large-scale metabolomic analysis are publicly available in the Mendeley database (http://dx.doi.org/10.17632/nxzs4dtztg.1; https://data.mendeley.com/datasets/nxzs4dtztg/2). Raw metabolomic data from small-scale metabolomic analysis are publicly available in the Mendeley database (http://dx.doi.org/10.17632/nxzs4dtztg.1; https://data.mendeley.com/datasets/nxzs4dtztg/2).

Research involving human participants, their data, or biological material

Policy information about studies with human participants or human data. See also policy information about sex, gender (identity/presentation), and sexual orientation and race, ethnicity and racism.

Reporting on sex and gender	n/a
Reporting on race, ethnicity, or other socially relevant groupings	n/a

Population characteristics	n/a
Recruitment	n/a
Ethics oversight	All animal experiments were performed after approval by the NYUAD-IACUC (Protocol 21-0005).

Note that full information on the approval of the study protocol must also be provided in the manuscript.

Field-specific reporting

Please select the one below that is the best fit for your research. If you are not sure, read the appropriate sections before making your selection.

☒ Life sciences ☐ Behavioural & social sciences ☐ Ecological, evolutionary & environmental sciences

For a reference copy of the document with all sections, see nature.com/documents/nr-reporting-summary-flat.pdf

Life sciences study design

All studies must disclose on these points even when the disclosure is negative.

Sample size	No sample size calculations were performed and number of replicates were based on standard practices in the field. Hi-C, ATAC-Seq and ChIP-Seq experiments were performed using two replicates. RNA seq was performed using 4 replicates. All other experiments were done at least in triplicates in two separate experiments or otherwise stated in Material and Methods part.
Data exclusions	no data were excluded
Replication	Sequencing experiments were performed using at least two replicates to ensure reproducibility. All other experimetns were performed at least in triplicates. all replication attempts were succesful
Randomization	Randomization was not relevant to the study as we did blinding of the samples for data collection and analysis
Blinding	Blinding was performed for the majority of the experiments.

Reporting for specific materials, systems and methods

We require information from authors about some types of materials, experimental systems and methods used in many studies. Here, indicate whether each material, system or method listed is relevant to your study. If you are not sure if a list item applies to your research, read the appropriate section before selecting a response.

Materials & experimental systems

n/a	Involved in the study	
☐	☒	Antibodies
☐	☒	Eukaryotic cell lines
☒	☐	Palaeontology and archaeology
☐	☒	Animals and other organisms
☒	☐	Clinical data
☒	☐	Dual use research of concern
☒	☐	Plants

Methods

n/a	Involved in the study	
☐	☒	ChIP-seq
☒	☐	Flow cytometry
☒	☐	MRI-based neuroimaging

Antibodies

Antibodies used	Antibodies against GAPDH (ab8245), The Total OXPHOS Rodent WB Antibody Cocktail kit (ab110413), H3K9Ac (ab4441), H3K4me3 (ab8580), HA-tag (ab9110), Snf2h (ab3749), Alexa Fluor 555 Goat Anti-Rabbit (ab150078), Horseradish peroxidase (HRP)-fused Goat Anti-Rabbit (ab6721) and Rabbit Anti-Mouse (ab6728) were purchased from Abcam. Antibodies against TFAM (ABE483) and Mct1 (AB3538P) were purchased from Merc Millipore. Antibody against EGFR (2-18C9) was obtained from Agilent. The anti-Hif1a (D1S7W) antibody was obtained from Cell signaling. The anti-beta actin (A5316) antibody was obtained from Sigma. The anti-NM1 antibody was described and characterized earlier (Venit 2020). Where applicable, all antibodies were used according to manufacturers' protocols. Primary antibodies were diluted 1:200 and secondary antibodies 1:400 in 1x PBST buffer with 3% bovine serum albumin for immunofluorescent experiments, and 1:1000 and 1:2000 respectively in 1xTBST buffer for western blot analysis.
Validation	all Validations can be found on the manufacturer's webpages:<br>GAPDH (ab8245) - https://www.abcam.com/products/primary-antibodies/gapdh-antibody-6c5-loading-control-ab8245.html<br>The Total OXPHOS Rodent WB Antibody Cocktail kit (ab110413) - https://www.abcam.com/products/panels/total-oxphos-rodent-wb-antibody-cocktail-ab110413.html -<br>H3K9Ac (ab4441) - https://www.abcam.com/products/primary-antibodies/histone-h3-acetyl-k9-antibody-chip-grade-ab4441.html

H3K4me3 (ab8580) - https://www.abcam.com/products/primary-antibodies/histone-h3-tri-methyl-k4-antibody-chip-grade-ab8580.html
HA-tag (ab9110) - https://www.abcam.com/products/primary-antibodies/ha-tag-antibody-chip-grade-ab9110.html
Snf2h (ab3749) - https://www.abcam.com/products/primary-antibodies/snf2h-antibody-chip-grade-ab3749.html
Alexa Fluor 555 Goat Anti-Rabbit (ab150078) - https://www.abcam.com/products/secondary-antibodies/goat-rabbit-igg-hl-alexa-fluor-555-ab150078.html
Horseradish peroxidase (HRP)-fused Goat Anti-Rabbit (ab6721) - https://www.abcam.com/products/secondary-antibodies/goat-rabbit-igg-hl-hrp-ab6721.html
Horseradish peroxidase (HRP)-fused Rabbit Anti-Mouse (ab6728) - https://www.abcam.com/products/secondary-antibodies/rabbit-mouse-igg-hl-hrp-ab6728.html
TFAM (ABE483) - https://www.merckmillipore.com/INTL/en/product/Anti-TFAM-Antibody,MM_NF-ABE483?ReferrerURL=https%3A%2F%2Fwww.google.com%2F
Mct1 (AB3538P) - https://www.merckmillipore.com/INTL/en/product/Anti-Monocarboxylate-Transporter-1-Antibody,MM_NF-AB3538P?ReferrerURL=https%3A%2F%2Fwww.google.com%2F&bd=1
Hif1a (D1S7W) - https://www.cellsignal.com/products/primary-antibodies/hif-1a-d1s7w-xp-rabbit-mab/36169?_requestid=2833183

Eukaryotic cell lines

Policy information about cell lines and Sex and Gender in Research

Cell line source(s): Nuclear Myosin 1 Knock-Out (NM1 KO) cell lines were derived from wild-type mouse embryonic fibroblasts (MEFs) (ATCC® CRL-2752) (NM1 WT) using the CRISPR/Cas9 system (Venit, 2020). For the NM1 rescue experiment we transduced NM1 KO MEFs by lentiviral vector carrying coding sequence for NM1 fused with HA and V5 tag (VectorBuilder). The cells expressing NM1-HA-V5 were selected by Neomycin and single clones tested by Western blot by specific anti-HA antibodies.

Authentication: the expression of NM1 in each cell line was tested by western blot and is part of the manuscript. In short, Antibodies against NM1 protein and antibodies against Ha-tag were used to specify the cell line.

Mycoplasma contamination: Cells were tested negative for mycoplasma contamination.

Commonly misidentified lines (See ICLAC register): no commonly misidentified lines were used in the study.

Animals and other research organisms

Policy information about studies involving animals; ARRIVE guidelines recommended for reporting animal research, and Sex and Gender in Research

Laboratory animals: Female Balb/c nude mice (16 weeks old; 25-35 g in weight) were purchased from Jackson Laboratory, USA. The mice were housed in a pathogen-free sterile ventilation system supplied with sterile woodchip and ad libitum feed (PicoLab Rodent Diet 20, 5053) and water supply. Mice were kept in a room maintained at a temperature of 21-24 °C on a schedule of 12 h light/dark cycle. All animal experiments were performed after approval by the NYUAD-IACUC (Protocol 21-0005).

Wild animals: no wild animals were used in the study

Reporting on sex: Female mice were used in the study

Field-collected samples: no field-collected samples were used

Ethics oversight: All animal experiments were performed after approval by the NYUAD-IACUC (Protocol 21-0005).

Note that full information on the approval of the study protocol must also be provided in the manuscript.

Plants

Seed stocks: n/a

Novel plant genotypes: n/a

Authentication: n/a

ChIP-seq

Data deposition

☒ Confirm that both raw and final processed data have been deposited in a public database such as GEO.

☒ Confirm that you have deposited or provided access to graph files (e.g. BED files) for the called peaks.

Data access links
May remain private before publication.

https://www.ncbi.nlm.nih.gov/geo/query/acc.cgi?acc=GSE202716

Files in database submission

WT-SNF2H-Rep2_S14_read2.fastq.gz
WT-SNF2H-Rep2_S14_read1.fastq.gz
WT-SNF2H-Rep1_S13_read2.fastq.gz
WT-SNF2H-Rep1_S13_read1.fastq.gz
WT-Input2-1-10conc_S5_read2.fastq.gz
WT-Input2-1-10conc_S5_read1.fastq.gz
WT-Input1-1-10conc_S4_read2.fastq.gz
WT-Input1-1-10conc_S4_read1.fastq.gz
WT-H3K9me3-Rep2_S9_read2.fastq.gz
WT-H3K9me3-Rep2_S9_read1.fastq.gz
WT-H3K9me3-Rep1_S8_read2.fastq.gz
WT-H3K9me3-Rep1_S8_read1.fastq.gz
WT-H3K9me-Rep2_S11_read2.fastq.gz
WT-H3K9me-Rep2_S11_read1.fastq.gz
WT-H3K9me-Rep1_S10_read2.fastq.gz
WT-H3K9me-Rep1_S10_read1.fastq.gz
WT-H3K9ac-Rep2_S13_read2.fastq.gz
WT-H3K9ac-Rep2_S13_read1.fastq.gz
WT-H3K9ac-Rep1_S12_read2.fastq.gz
WT-H3K9ac-Rep1_S12_read1.fastq.gz
WT-H3K27me3-Rep2_S16_read2.fastq.gz
WT-H3K27me3-Rep2_S16_read1.fastq.gz
WT-H3K27me3-Rep1_S15_read2.fastq.gz
WT-H3K27me3-Rep1_S15_read1.fastq.gz
WT-H3K27ac-Rep2_S18_read2.fastq.gz
WT-H3K27ac-Rep2_S18_read1.fastq.gz
WT-H3K27ac-Rep1_S17_read2.fastq.gz
WT-H3K27ac-Rep1_S17_read1.fastq.gz
NM1-KO-SNF2H-Rep2_S21_read2.fastq.gz
NM1-KO-SNF2H-Rep2_S21_read1.fastq.gz
NM1-KO-SNF2H-Rep1_S20_read2.fastq.gz
NM1-KO-SNF2H-Rep1_S20_read1.fastq.gz
NM1-KO-H3K9me3-Rep2_S17_read2.fastq.gz
NM1-KO-H3K9me3-Rep2_S17_read1.fastq.gz
NM1-KO-H3K9me3-Rep1_S16_read2.fastq.gz
NM1-KO-H3K9me3-Rep1_S16_read1.fastq.gz
NM1-KO-H3K9ac-Rep2_S21_read2.fastq.gz
NM1-KO-H3K9ac-Rep2_S21_read1.fastq.gz
NM1-KO-H3K9ac-Rep1_S20_read2.fastq.gz
NM1-KO-H3K9ac-Rep1_S20_read1.fastq.gz
NM1-KO-H3K4me-Rep2_S19_read2.fastq.gz
NM1-KO-H3K4me-Rep2_S19_read1.fastq.gz
NM1-KO-H3K4me-Rep1_S18_read2.fastq.gz
NM1-KO-H3K4me-Rep1_S18_read1.fastq.gz
NM1-KO-H3K27me3-Rep2_S23_read2.fastq.gz
NM1-KO-H3K27me3-Rep2_S23_read1.fastq.gz
NM1-KO-H3K27me3-Rep1_S22_read2.fastq.gz
NM1-KO-H3K27me3-Rep1_S22_read1.fastq.gz
NM1-KO-H3K27ac-Rep2_S2_read2.fastq.gz
NM1-KO-H3K27ac-Rep2_S2_read1.fastq.gz
NM1-KO-H3K27ac-Rep1_S1_read2.fastq.gz
NM1-KO-H3K27ac-Rep1_S1_read1.fastq.gz
KO-Input2-1-10conc_S7_read2.fastq.gz
KO-Input2-1-10conc_S7_read1.fastq.gz
KO-Input1-1-10conc_S6_read2.fastq.gz
KO-Input1-1-10conc_S6_read1.fastq.gz
GSE202716_KO_H3K27ac.bw
GSE202716_KO_H3K27me3.bw
GSE202716_KO_H3K4me.bw
GSE202716_KO_H3K9ac.bw
GSE202716_KO_H3K9me3.bw
GSE202716_KO_Input.bw
GSE202716_KO_SNF2H.bw
GSE202716_WT_H3K27ac.bw
GSE202716_WT_H3K27me3.bw
GSE202716_WT_H3K4me.bw
GSE202716_WT_H3K9ac.bw
GSE202716_WT_H3K9me3.bw
GSE202716_WT_Input.bw
GSE202716_WT_SNF2H.bw

Genome browser session
(e.g. UCSC)

n/a

Methodology

Replicates

2 replicates per condition were used

Sequencing depth

Sample/Mapped Reads/Length/Type
KO_1_H3K27ac/40515165/75/Paired-end
KO_1_H3K27me3/27482202/75/Paired-end
KO_1_H3K4me/31944961/75/Paired-end
KO_1_H3K9ac/33916637/75/Paired-end
KO_1_H3K9me3/27865982/75/Paired-end
KO_1_Input/36945600/75/Paired-end
KO_1_SNF2H/32624879/75/Paired-end
WT_1_H3K27ac/28871060/75/Paired-end
WT_1_H3K27me3/28531128/75/Paired-end
WT_1_H3K4me/32044255/75/Paired-end
WT_1_H3K9ac/36881910/75/Paired-end
WT_1_H3K9me3/28673413/75/Paired-end
WT_1_Input/39608550/75/Paired-end
WT_1_SNF2H/28356663/75/Paired-end
KO_2_H3K27ac/36319308/75/Paired-end
KO_2_H3K27me3/32246869/75/Paired-end
KO_2_H3K4me/33361608/75/Paired-end
KO_2_H3K9ac/30856481/75/Paired-end
KO_2_H3K9me3/32578605/75/Paired-end
KO_2_Input/40559012/75/Paired-end
KO_2_SNF2H/23011140/75/Paired-end
WT_2_H3K27ac/30507627/75/Paired-end
WT_2_H3K27me3/27642212/75/Paired-end
WT_2_H3K4me/46448374/75/Paired-end
WT_2_H3K9ac/36835608/75/Paired-end
WT_2_H3K9me3/33260979/75/Paired-end
WT_2_Input/45107413/75/Paired-end
WT_2_SNF2H/40213255/75/Paired-end

Antibodies

H3K9Ac (ab4441), H3K4me3 (ab8580), Snf2h (ab3749)

Peak calling parameters

Peaks were called using macs2 for both ChIP-Seq and ATAC-Seq. Parameters used are specified in the relevant methods section.

Data quality

Correlation between replicates was analyzed with deeptools. Peaks were called using macs2 FDR cutoff of 0.05.

Software

ChIP-Seq data was processed with trimmomatic, bwa_mem, samtools, and picard and analyzed using HOMER, Macs2 and Deeptools. Detailed pre-processing, alignment and analysis steps are described in the relevant methods sections.
